# Host Plant Variation and Lack of Genetic Differentiation in Populations of *Dione* (*Agraulis*) *dodona* Lamas & Farfán (Lepidoptera: Nymphalidae)

**DOI:** 10.3390/insects13090819

**Published:** 2022-09-08

**Authors:** Jackie Farfán, José Cerdeña, Wilson Huanca-Mamani, Héctor A. Vargas, Gislene L. Gonçalves, Gilson R. P. Moreira

**Affiliations:** 1 PPG Biologia Animal, Departamento de Zoologia, Instituto de Biociências, Universidade Federal do Rio Grande do Sul, Avenue Bento Gonçalves 9500, Porto Alegre 91501-970, RS, Brazil; 2 Museo de Historia Natural, Universidad Nacional de San Agustín de Arequipa, Avenue Alcides Carrión s/n, Arequipa 04000, Peru; 3 Departamento de Producción Agrícola, Facultad de Ciencias Agronómicas, Universidad de Tarapacá, Arica 1000000, Chile; 4 Departamento de Recursos Ambientales, Facultad de Ciencias Agronómicas, Universidad de Tarapacá, Casilla 6-D, Arica 1000000, Chile; 5 Departamento de Genética, Instituto de Biociências, Universidade Federal do Rio Grande do Sul, Avenue Bento Gonçalves 9500, Porto Alegre 91501-970, RS, Brazil; 6 Departamento de Zoologia, Instituto de Biociências, Universidade Federal do Rio Grande do Sul, Avenue Bento Gonçalves 9500, Porto Alegre 91501-970, RS, Brazil

**Keywords:** microsatellites, mitochondrial DNA, Heliconiinae, Andes

## Abstract

**Simple Summary:**

In this study, we analyzed the population genetics of *Dione* (*A.*) *dodona* Lamas and Farfán (Nymphalidae), a recently described butterfly from xerophytic environments of the western slopes of the Central Andes, associated with *Malesherbia* *tenuifolia* (Passifloraceae). Searches for additional host-plant species were carried out throughout its known distribution. In total, seven species of *Malesherbia* have been reported as host plants for *D*. (*A.*) *dodona*, extending its distribution as far north as 50 km. We obtained samples of the last larval instar in six localities on different host-plant species that have disjointed distributions. We inferred the genetic diversity and structure between populations by using microsatellites and mitochondrial COI markers. The results showed a weak genetic differentiation between the evaluated populations, suggesting a continuum of gene flow through high dispersal, along with the disjoint occurrence of the host plants.

**Abstract:**

*Dione* (*Agraulis*) *dodona* (Nymphalidae: Heliconiinae) is a butterfly restricted to the western slopes of the Andes of Peru and Chile and is associated with *Malesherbia tenuifolia* in xerophytic environments. In this study, we found six additional species of host plants for *D.* (*A.*) *dodona* belonging to the genus *Malesherbia* (Passifloraceae). We used mitochondrial DNA sequences (COI) and microsatellites to screen genetic variation and investigate population structure in six geographic disjointed populations of *D*. (*A*.) *dodona* associated with distinct *Malesherbia* species. The PCoA analysis based on allele frequencies evidenced a lack of differentiation among populations and a low *F*_ST_. The Bayesian cluster analyses revealed the existence of three genetically distinct groups, but almost all individuals present an admixture ancestry. An absence of isolation by distance pattern was observed. Possible scenarios are discussed: a bottleneck or recent colonization from interconnected populations from the south, and ongoing gene flow among local populations by high dispersal through a landscape formed of isolated populations of *Malesherbia*.

## 1. Introduction

The Neotropical butterfly genus *Dione* Hübner (Nymphalidae: Heliconiinae) includes a newly described species, *Dione (Agraulis) dodona* Lamas & Farfán, restricted to xeric slopes of the Andes in Peru and Northern Chile, associated with the host plant *Malesherbia tenuifolia* D. Don (Passifloraceae) [1]. The females of *D. (A.) dodona* oviposit on flowers or leaves near them, a behavior that is unique among Heliconiines, because the first instar feed on the internal structures of the flowers [1]. *Malesherbia* R. and P. is a genus distributed in the Central and Southern Andes in Peru, Chile, and Argentina, mostly in the lower parts of the western slopes on rocky and sandy soils, in arid and semi-arid habitats [2,3,4,5]. The subgeneric classification of *Malesherbia* comprises five sections, of which only the *Malesherbia* section is found in Peru and Northern Chile, with *M. tenuifolia* being a member, along with thirteen other species [6], mostly known from single localities and with discontinuous or isolated distributions [4,5,6]. The geographic range of *D*. (*A.*) *dodona* appears to be narrowly based on the description records besides the type locality; this butterfly has been considered rare [1,7], with adults collected only in two Regions in Northern Chile and four Departments in Peru, with about 1400 km between the most distant records [1].

As the northernmost occurrence of *D*. (*A.*) *dodona* in the Peru–Chile desert [1] exceeds the northern limit of its only documented host plant, *M. tenuifolia* [4], and the presence of additional populations of several species of *Malesherbia* between the Pacific Coast and the Andes of South–Central Peru and Northern Chile provides a potential for (i) wider geographic and potential additional host-plant ranges for this butterfly and (ii) the occurrence of isolated populations due to a discontinuous distribution of host plants. Accordingly, to verify the entire occurrence of *D. (A.) dodona* (considered a rare species) and to assess its host-plant range, it should be screened in habitats where *Malesherbia* species occur. Considering the patchy distribution of *Malesherbia* throughout extensive arid areas in the desert landscape of Western South–Central Peru and Northern Chile, together with the highly specialized egg-laying-site selection of the females, some degree of genetic differentiation might be expected for putative populations of *D. (A.) dodona*.The scale at which intraspecific gene flow occurs is an important property of a species, with implications for conservation. However, the spatial scale of genetic connectivity is often unknown since its assessment either requires large-scale mark-recapture studies or genetic data [8]. A simple marker used to infer genetic variation is the mitochondrial DNA (mtDNA) due to its suitable rate of mutation. However, the nature of inheritance (strictly maternal) and absence of recombination limit its use for gene-flow estimates. A more appropriate marker for population genetics and conservation studies is the microsatellites, given their high variability, biparental inheritance, and co-dominance [9,10]. However, microsatellites have been not widely used in studies of lepidopterans because of a paucity of these markers in their genomes [11]; that is, they are difficult and costly to develop. In Heliconiini, microsatellites have been developed for three species, two belonging to the genus *Heliconius* [12,13] and one to *Dione* [14]. Since cross-amplification was successful for *Dione (Agraulis) vanillae* [14], we consider it practicable to amplify polymorphic loci in populations of *D. (A.) dodona*.

In phytophagous insects, the distribution of intraspecific genetic diversity can be determined by the intrinsic and extrinsic factors of the organism. Life history can be of significant importance [15], but environmental factors mostly promote the relative isolation of populations, such as geographic distances, physical barriers to gene flow, habitat suitability and/or fragmentation, and the use of host plants [16]. Particularly, the host-plant fidelity has been demonstrated to play a major role in genetic isolation between populations [17].

**Figure 1:**
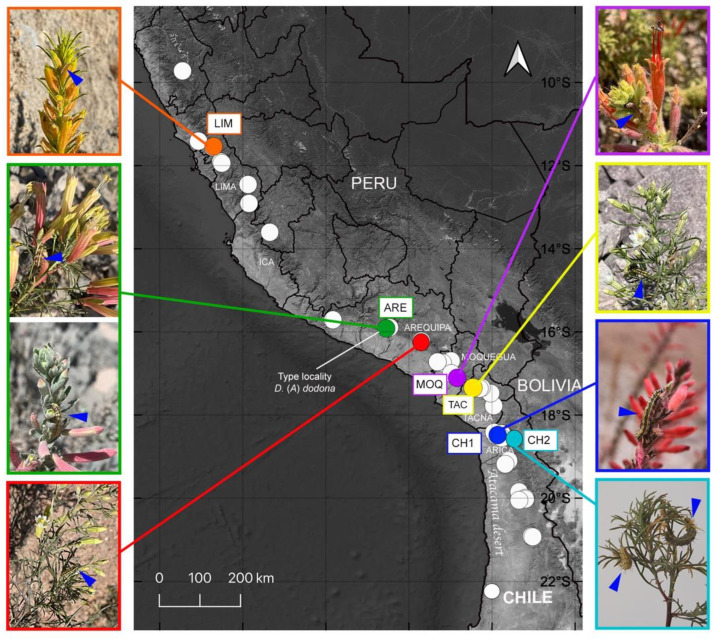
Distribution of *Malesherbia* taxa section *Malesherbia* (white dots) with sampling sites of larvae of *D*. (*A.*) *dodona* (colored dots) on western slopes of Andes. Orange = Lima (LIM); green = Arequipa (type locality) (ARE); red = Arequipa (Yura); purple = Moquegua (MOQ); yellow = Tacna (TAC); blue = Chile 1 (CH1); light blue = Chile 2 (CH2). The host plants depicted in each population of *D*. (*A.*) *dodona* demonstrate variability observed in the xerophytic environments (see Supplementary Materials Table S1 for details of host plants’ localities). Flowers of host plants with fifth instar larvae (blue close arrow) are shown on both sides of the map.

Considering that the distribution of *D. (A.) dodona* [1] is much wider (ca. 1200 km) than that of *M. tenuifolia* [3,5], one would expect this butterfly is not monophagous but feeds on additional host plants. Several *Malesherbia* species were recorded by Beltrán et al. [4] for the area, but such plants have not been searched regarding usage by this butterfly. Thus, in this work, we studied the geographic and host-plant ranges of *D. (A.) dodona* and evaluated the levels of genetic differentiation between populations along the corresponding latitudinal gradient in the western slopes of the Central Andes (Figure 1).

## 2. Materials and Methods

### 2.1. Sample Collection and Host-Plant Records

Fieldwork was performed from November 2020 to May 2022, screening for *Malesherbia* plants along the roadways, covering a latitudinal gradient of seven degrees (∼1200 km), from Central Peru to Northern Chile, on western slopes of the Andes, between 1000 and 3000 m elevation (Figure 1). An intensive search for immature stages of *D.* (*A*.) *dodona* on flowers of *Malesherbia* spp. was carried out in each locality where plants were found. Larvae of the fifth instar of *D*. (*A.*) *dodona* were collected and preserved in 70% ethanol. Samples of *Malesherbia* from each locality were also collected and identified at the species level, based on the literature [3,4,5].

### 2.2. Genetic Analysis

**Figure 2:**
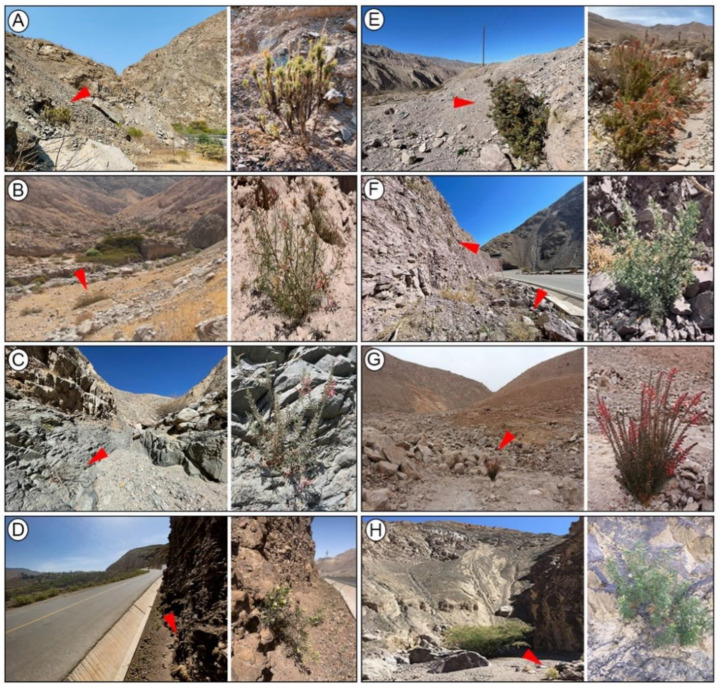
Locality and host-plant records (*Malesherbia* spp.) for *D.* (*A.*) *dodona* on western slopes of Andes. (**A**) Peru, Lima, Canta, *M. tubulosa*. (**B**) Peru, Arequipa, Pacaychacra (type locality), *M. tenuifolia*. (**C**) Peru, Arequipa, Pacaychacra (type locality), *M. fatimae*. (**D**) Peru, Arequipa, Yura, *M. angustisecta*. (**E**) Peru, Moquegua, Torata, *M. ardens*. (**F**) Peru, Tacna, Chululuni, *M. arequipensis*. (**G**) Chile, Arica, Cuesta El Águila, *M. auristipulata*. (**H**) Chile, Arica, Las Peñas, *M. tenuifolia*. From right to left column: Panoramic view of locality (red close arrow points to larval hostplant); host-plant species. Detailed location information is described in Supplementary Materials Table S1.

Six localities identified with the presence of *D*. (*A.*) *dodona* (*n* = number of individuals sampled) feeding on flowers of *Malesherbia* spp. were used for population genetics analysis: (1) LIM—Peru, Lima Department (*n* = 8), Canta; (2) ARE—Peru, Arequipa Department (*n* = 10), Pacaychacra, type locality; (3) MOQ—Peru, Moquegua Department (*n* = 10), Torata; (4) TAC—Peru, Tacna Department (*n* = 10), Chululuni; (5) CH1—Chile, Arica Region (*n* = 9), Cuesta El Águila; and (6) CH2—Chile, Arica Region (*n* = 5), Las Peñas (Figure 1 and Figure 2 and Supplementary Materials Table S1).

#### 2.2.1. DNA Extraction, Polymerase Chain Reaction Amplification, and Sequencing

Genomic DNA was isolated from each specimen, using larvae tissue stored in ethanol 70%, according to Huanca-Mamani et al. [18]. Genomic DNA was sent to Macrogen Inc. (Seoul, South Korea) for PCR amplification, purification, and sequencing of the mitochondrial gene Cytochrome oxidase subunit 1 (COI), using primers HCO2198 and LCO1490 with conditions described by Folmer et al. [19]. Sequence alignments were performed by using CodonCode Aligner (CodonCode Corp., Centerville, OH, USA) and inspected manually.

We surveyed 14 microsatellites developed in the genus *Dione*, which also proved to be polymorphic in the subgenus *Agraulis* [20]. An M13 tail (5′-TGTAAAACGACGGCCAGT-3′) was linked at the 5′ of the forward primer of each polymorphic loci. A primer mix consisting of M13 labeled with a fluorescent dye (6-FAM, HEX, or TAMRA) was used for PCR at each locus. Each PCR reaction contained 50 ng of DNA, 1X buffer, 2.5 mM MgCl_2_, 0.2 μM each dNTP, 0.25 μM forward and 0,25 μM M13 fluorescently labeled primers, 0.5 μM reverse primer, and 1 unit of Taq DNA polymerase (Thermo Scientific, Waltham, MA, USA). PCRs were performed by using the following conditions: 95 °C for 2 min, followed by 35 cycles of 30 s of 94 °C, specific annealing temperature for 30 s, 1 min of 72 °C, and a final extension of 72 °C for 4 min [14]. All individuals were genotyped (multiplexed by using different fluorescence and size ranges), using a Liz GeneScan 600 (Life Technologies, Carlsbad, CA, USA) standard marker of molecular weight in an ABI Genome Analyzer by the commercial facility of Macrogen.

#### 2.2.2. Data Analysis

Genetic diversity within each population was estimated by using the mean number of alleles per locus (allelic richness (A)), the percentage of polymorphic loci (%P), observed heterozygosity (H_O_), and the heterozygosity expected from Hardy–Weinberg equilibrium (H_E_) [21], using the software GenAlEx v 6.5 [22]. Genotypes were checked for the presence of null alleles, using the software Micro-Checker 2.2 [23]. Linkage disequilibrium and deviations from the Hardy–Weinberg equilibrium [24] were tested by using ARLEQUIN 3.5 [25]. Sequential Bonferroni corrections were applied to correct for multiple comparisons [26], with *p* = 0.05 to adjust the statistical significance levels. Standard genetic diversity indices, such as the number of COI haplotypes and haplotype diversity per population, were estimated for mtDNA sequences, using ARLEQUIN 3.5. The topological relationship between haplotypes was estimated by using the median-joining approach [27] implemented in NETWORK 4.1.

Genetic differentiation between the six populations was characterized by exact tests [28] and by estimating pairwise *F*_ST_ [29], within ARLEQUIN 3.5. The levels of significance for multiple tests were adjusted by the sequential Bonferroni method [26]. We investigated whether inter-population genetic distances (based on microsatellite loci) increased linearly with geographic distance. Thus, genetic (*F*_ST_) and geographic (km, linear) distances between populations were calculated in GenAlEx 6.5 and Google Earth 7.3, respectively. Pairwise genetic and geographic distance matrices were correlated by using a Mantel test, with a test for a significant relationship by 9999 random permutations that was also implemented in GenAlEx 6.5. To investigate the existence of hierarchical levels of population structure, an analysis of molecular variance (AMOVA) was performed by using allele frequencies with ARLEQUIN 3.5, following a geographic pattern of north (LIM, ARE, MOQ, and TAC) to south (CH1 and CH2) structure.

As an alternative approach to represent the genetic relationship among the six populations of *D. (A.) dodona*, a principal component analysis (PCoA) was applied by using gene frequencies of all six variable microsatellite loci. The frequencies of all the alleles at a single locus were considered independent variables. Accordingly, correlation matrices were computed from the gene frequencies of all the loci, as well as the eigenvalues of all the principal components, the proportions of individual eigenvalues to the total variance (contribution rates of components), and the raw scores of every individual for each of the principal components. PCoA was performed by using the program PCAGEN 1.2.1.

A Bayesian clustering procedure implemented in STRUCTURE v 2.3.3 [30] was used to infer the number of distinct genetic populations represented in our samples. The number of *K* clusters was set from one to six (i.e., the total number of sampled populations). MCMC simulations were performed by using the admixture model and correlated genetic frequencies among individuals. Simulations ran for 1,000,000 iterations, using 100,000 iterations as a burn-in period. We evaluated the length of burn-in, as well as the stabilization of the parameters of the analysis, using the plot function present in STRUCTURE 2.3.4 software. Then we ran results through STRUCTURE HARVESTER (https://taylor0.biology.ucla.edu/structureHarvester/, accessed on 1 May 2022) [31], using the maximum delta *K* as the selected value of *K* (Evanno method) [32].

Finally, we looked for a genetic signature of past demographic events (e.g., population expansion or decline) in populations of *D. (A.) dodona* by using COI sequences. We performed Fu’s *F*s test by comparing *F*s statistics against a distribution generated from 1000 random samples, using Arlequin v.3.5. The statistic *F*s tend to be negative under an excess of recent mutations, and a significant negative value was then taken as evidence of population growth. We also estimated Tajima’s *D* statistics, and the significance of the *D* statistic was tested by generating random samples under the hypothesis of selective neutrality and population equilibrium, using a coalescent algorithm. A mismatch distribution analysis was also performed for the species, using the population growth–decline model in the software DNAsp 6.12.03 [33].

## 3. Results

### 3.1. Host-Plant Variation

Seven host-plant species for *D. (A.) dodona* were recorded in seven localities (including the type locality of *D. (A.) dodona*) on the western slopes of the Andes in Peru and Chile (Figure 1 and Supplementary Materials Table S1). Six correspond to new records: *Malesherbia angustisecta* Harms, *M. arequipensis* Ricardi, *M. ardens* J.F. Macbr., *M. auristipulata* Ricardi, *M. fatimae* H. Beltrán and *M. Weigend*, and *M. tubulosa* (Cav.) J. St.-Hil. Each of the seven localities presented a distinct species of *Malesherbia*, except for CH2, which showed *M. tenuifolia*, the same species recorded in type locality (ARE). An additional species, *M. fatimae,* was found in the population of *D. (A.) dodona* of the type locality. The environments where *Malesherbia* spp. occurs correspond to xerophytic slopes or dry ravines with very little vegetation (Figure 2).

A population of *D*. (*A.*) *dodona* (LIM) was recorded 50 km north of the northernmost known record of the species; therefore, we expanded its distribution to the north in the Department of Lima, in the Chillon river basin in the locality of Canta, with immature stages associated with *M. tubulosa*. Additionally, two *Malesherbia* species were found in two localities without immature stages of *D*. (*A.*) *dodona*: *M. laraosensis* H. Beltrán and M. Weigend (Peru, Lima Department, Yauyos, 2800 m); and *M. tenuifolia* (Peru, Arequipa Department, Cotahuasi, 2400 m).

### 3.2. Genetic Differentiation

Eight of the fourteen microsatellite loci were correctly amplified for all 52 individuals; six of them were polymorphic (Supplementary Materials Table S2). Exact tests of genotypic linkage disequilibrium (either global or for populations) yielded no significant values (*p* = 0.05), thus suggesting that loci are independent. Significant positive departures from the Hardy–Weinberg equilibrium were found in two loci (Dione9 and Dione27) for two populations (LIM and CH2) (Supplementary Materials Table S3). Using Micro-Checker 2.2, we detected a signal for null alleles at these two loci, but there was no indication of errors caused by stutter bands or large allele dropout. Values for allelic richness, polymorphic loci (%), and observed and expected heterozygosities for each population are given in Supplementary Materials Table S4.

As two of our six loci were possibly affected by null alleles, we performed all statistics twice: once for all loci, and a second time for only the four loci without null allele signals. In the following analysis, we only show the values based on all six loci (the results based on four loci are given in Supplementary Materials Table S5). Because all analyses based on the four loci without null allele signals (i.e., genetic diversities and structures) showed similar results to those based on all loci, we conclude that the result is reliable population-genetic and phylogeographic information that has not been blurred by null alleles.

**Figure 3:**
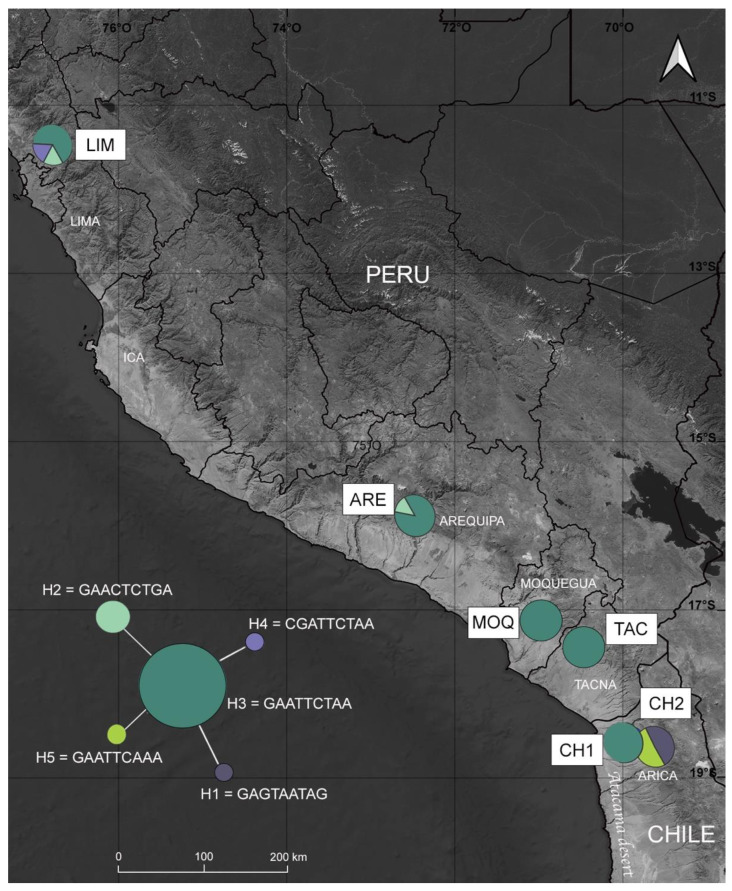
Map showing mtDNA haplotype distribution of *Dione (Agraulis) dodona*. Median-joining network for COI haplotypes appears in the lower-left corner, with the size of the circles proportional to frequency (1 [H1, H4 and H5), 2 [H2] and 29 [H3]).

In the alignment of 654 bp of COI gene, we found nine variable sites (Supplementary Materials Table S6). All the observed polymorphisms were single base-pair mutations; five were transitions and four were transversions. The haplotype diversity values range from 0.22 to 1, with an average value of 0.28, and nucleotide diversity ranged from 0 to 0.005, with a global value of 0.007 (Supplementary Materials Table S4). In total, we identified five haplotypes (Figure 3 and Supplementary Materials Table S4). The median-joining network was star-shaped, in that some less-frequent haplotypes (H1, H4, and H5) were closely related to a single common haplotype (H3). The haplotype H3 was shared between all populations, except for CH2. LIM presented three haplotypes (H2, H3, and H4), and CH2 showed two haplotypes (H1 and H5).

**Figure 4:**
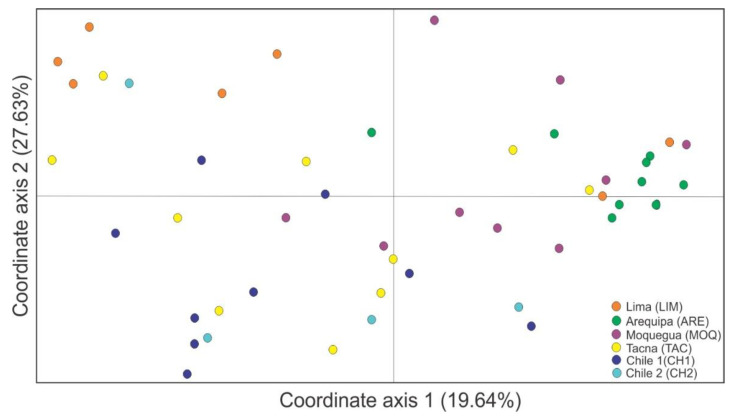
Scores of a principal component analysis for the two leading axes based on allelic frequencies of microsatellites in six populations of *Dione (Agraulis) dodona*. Each color refers to a specific population.

Patterns of genetic differentiation were represented by a multivariate PCoA plot (Figure 4). The PCoA individual scores plotted into the first two principal component axes, coordinate axis 1 and coordinate axis 2, explained 19.64% and 27.63%, respectively, of the total genetic diversity.

**Figure 5:**
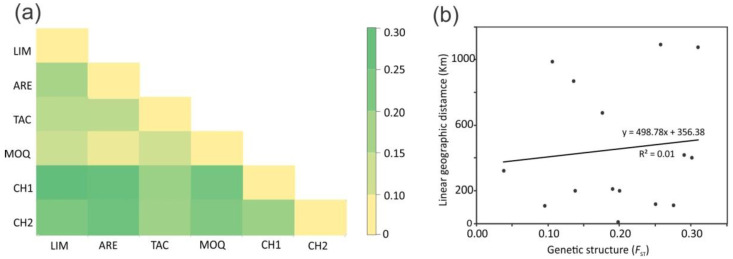
(**a**) Pairwise *F*_ST_ heatmap based on *F*_ST_ values among the six *Dione* (*Agraulis*) *dodona* sampling sites; dark green colors reflect high genetic differentiation (>0.15), and lighter colors indicate low differentiation (<0.15). (**b**) Relationship between pairwise genetic distance (*F*_ST_) and the logarithm of Euclidian geographic distance (km) among pairs of sampling sites.

Samples presented a lot of mixture in the PCoA plot, without a clear segregation pattern for any population. These trends were confirmed by the AMOVA results. When looking at a possible structure by geographic distance (north vs. south), the differences among groups were not significant. Accordingly, 13.5% (*p* = 0.1241) of the total variance component was explained by the north (LIM + ARE + MOQ + TAC) and south (CH1 + CH2) groups of populations (see Table 1). The greater proportion of variation (74.7%, *p* = 0.000) was identified within populations. The *F*_ST_ based on allele frequencies between pairs of populations ranged from 0.10 to 0.30 (Figure 5a), with a mean *F*_ST_ = 0.19. Increased differentiation between north (LIM, ARE, MOQ, and TAC) and south (CH1 and CH2) groups were observed. However, Mantel tests did not show a significant correlation between geographic distance and estimates of gene flow, thus failing to support a pattern of isolation by distance (r^2^ = 0.01, *p* > 0.05) (Figure 5b).

**Figure 6:**
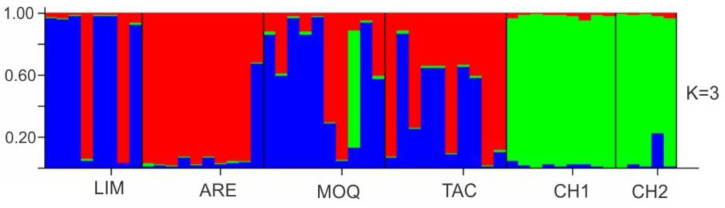
Structure analysis for *K* = 3, showing high admixture between individuals and an apparent increase in cluster 3 (green) toward southern populations.

A population structure analysis that was conducted by using the software Structure identified three Bayesian groups (*K* = 3) (Figure 6 and Supplementary Materials Figure S1). All three groups and all populations were found to share alleles, with slight evidence of two cluster formations, mainly composed of (i) LIM + ARE + MOQ + TAC and (ii) CH1 + CH2.

The results of Fu’s and Tajima’s neutrality tests for three populations of *D. (A.) dodona* (LIM, ARE, and MOQ, those that had enough variability for the analysis) are presented in Supplementary Materials Table S4. The tests of neutrality were not statistically significant for any populations. Similarly, the mismatch distribution suggests that these populations are in demographic equilibrium or decline (Supplementary Materials Figure S2).

## 4. Discussion

### 4.1. Evolution of Heliconiinae–Passifloraceae in the Andes

The evolutionary history of *Malesherbia* and *D. (A.) dodona* is intimate, both in space and time, considering the overlap in their distribution and origin of the clades (around 5 Mya) [7,34]. The increasing aridity of the Atacama Desert, associated with the Andes uplift, seems to have had an important effect on the flora [35,36,37]. Thus, the gradual isolation of species due to climatic fluctuations, hampered by geomorphologic events, may explain the geographic pattern found in *Malesherbia* [2]. From a geographic perspective, diversification of Heliconiinae is broadly tied to the area of occurrence of *D*. (*A*.) *dodona*. In fact, radiation of heliconiine butterflies occurred predominantly on the eastern slopes of the Andes in Colombia, Ecuador, and Peru, as well as in the upper/middle Amazon basin [38]. Thus, the Andes uplift may have had consequences on such a process, with the diversity of heliconiine butterflies resulting in being greater on the east side. The evolution of Heliconiinae is closely tied to Passifloraceae, the only plant family they use as a host. Benson et al. [39] were the first to point out that older lineages of Heliconiinae tend also to use older Passifloraceae as host plants, as in the case *D*. (*A*.) *dodona* larvae–*Malesherbia* plants. Such a co-evolutionary scenario predicts a progression from heliconiine species that feed on woody, long-lived, and presumably ancient food plants to those that specialize in herbaceous recent passion vines [39]. However, *Malesherbia* is a fragile herbaceous plant, not showing a scandent growing habitat. Furthermore, ecological opportunism and host and habitat shifts also seem to have played a major role in Heliconiinae speciation (for a review, see also References [40,41]). There is no record of additional herbivores associated with such plants in the area. The system provided by *D*. (*A*.) *dodona* and associated *Malesherbia* plants—which, in most cases, are geographically isolated—where populations of both seem to be very localized, restricted to single valleys on the western slopes of the Andes, offers a unique opportunity for future studies on heliconiine evolution of host-plant usage.

We found that *Dione* (*A.*) *dodona* uses seven distinct *Malesherbia* species as host plants, revealing it as an oligophagous species. This oligophagy was evidenced through the field collection, with each population presenting larvae that use distinct plants. Some of these individuals were brought to the laboratory and reared, exchanging the host plants. Flowers of *M. ardens* from Torata locality (MOQ) were offered to the first instar collected on *M. tenuifolia* from the type locality (ARE); the larvae accepted the flowers and fed during the following days without any negative behavior (unpublished data). All *Malesherbia* species reported here belong to the *Malesherbia* section, a lineage distributed from Northern Chile to Central Peru [6]; more than half of all the species in this section recorded for the Andean western slopes were used as host plants by *D*. (*A.*) *dodona* (7 vs. 13 spp.). Although the search for *Malesherbia* species during this study was restricted to the altitude range from 1000 to 3000 m elevation (due to the fact that all of these species in the study area are distributed between these altitudes), it is not ruled out that *D*. (*A.*) *dodona* can use as host plant *Malesherbia* section species that are found at lower elevations, as is the case of *M*. *tocopillana*, which is only known from its locality type (Tocopilla) in Chile, at an altitude range of 150 to 400 m elevation [5]; or also those species that could not be found during the intensive searches distributed on western slopes of the Andes, such as populations of *M*. *haemantha*, *M*. *scarlatiflora*, M. *splendens*, and *M*. *turbinea.*

The occurrence of *D*. (*A*.) *dodona* is strongly associated with the presence of *Malesherbia*, since all known records of adults match the distribution of this plant genus on the western slopes of the Central Andes [1]. Interestingly, the occurrence of *D*. (*A*.) *dodona* in a given area seems to relate to the availability of plant population belonging to the section *Malesherbia*, which is mostly allopatric. Changes in host plants among localities allow the occurrence of this butterfly in a vast area in the xeric western slopes of the Andes. However, the genus *Malesherbia* has a much wider distribution. Thus, in a broader sense, *D*. (*A*.) *dodona* may be limited in expansion due to the absence of *Malesherbia* species accepted as hosts, a subject which should be further explored. Heliconiinae, in general, is selective regarding intra- and inter-specific attributes of plants used as hosts, particularly regarding oviposition behavior and larval feeding, which may lead to local specializations [42,43,44,45]. Although most species are oligophagous and, thus, have relatively broad distributions since they alter hosts among localities, there is variation in this trait. Some have a narrower diet breath, using a few passion vines as hosts throughout their distribution range. For example, *Dione (Dione) moneta* and *Heliconius nattereri* are monophagous and, thus, have narrow geographic distributions in a given area of occurrence, which are limited by that of their host plants [39,42,46].

### 4.2. Genetic Connectivity through Suitable Areas

Our study shows that populations of *D. (A.) dodona* contain relatively little genetic variation and are not genetically subdivided. The overall haplotype and nucleotide diversity in the sister species *Dione (A.) vanillae* was 0.68 and 0.0143, respectively [47], which is quite higher than the 0.28 and 0.0009 found in *D. (A.) dodona*. Considering that the *D. (A.) vanillae* study included subspecies (further considered species) in addition to the population level, and that variation in sampling size and length of sequences likely influence such differences between studies, we looked to the microsatellites within the genus. Similarly, the other species of *Dione* showed little variation (13 alleles in 19 loci) [14].

It remains unknown whether *D*. (*A*.) *dodona* migrates or stays in a kind of dormancy when plants are not in active growth. Given that one congeneric species, *D. (D.) moneta*, is putatively migratory [48], we might consider that *D. (A.) dodona* could have a migratory potential. This aspect should be explored in conjunction with variation in phenology of *Malesherbia* species used as hosts, since females are highly selective regarding oviposition, laying eggs on flowers [1]. Thus, the little genetic variation of microsatellites observed can result from repeated localized extinctions and recolonizations, typical of a metapopulation structure [49]. Accordingly, patterns of variation found in *D. (A.) dodona* could result from lower historical population sizes and perhaps more recent colonization and/or bottleneck, as is supported by neutrality tests and mismatch distribution.

*Dione* (*Agraulis*) *dodona* is the oldest lineage among the extant Heliconiinae, ca. 8.59–2.8 My according to estimates of Nuñez et al. [7]; and several *Malesherbia* section species (e.g., *M*. *tenuifolia* and *M*. *arequipensis*) are relatively young (~5 My), with the genus *Malesherbia* having only recently colonized hyperarid habitats in deserts (~10 My) [34], thus possibly indicating that the population of *D*. (*A.*) *dodona* originated in the north of Chile and spread to Central Peru, together with the species of the *Malesherbia* section, being relatively recent colonization that does not allow us to detect genetic divergence between the populations. There exists a slight difference in the genetic structure about the southernmost population evaluated of *D*. (*A.*) *dodona* in the Arica region (CH2); however, this could be studied in greater detail in future studies including more southern populations.

In the second scenario, probably connectivity between the different populations of *D*. (*A.*) *dodona* (attested by low *F*_ST_ and admixture ancestry of individuals) is promoted by the distribution of the *Malesherbia* species. Historically, the species of *Malesherbia* recorded in Peru were considered isolated populations in their majority, due to the few records [4]. However, this may be largely due to a constraint in evaluating areas of difficult access on the western slopes, which resulted in gaps of information along with large areas, leading to the assumption that *Malesherbia* species were absent. An example is the Department of Ica, which connects ARE and LIM populations, where no *Malesherbia* species were recorded. However, probably due to the lack of access to areas where this plant genus grows, we assume that if a species of *Malesherbia* is recorded in the future in this Department, it could be the link between the populations of *D*. (*A.*) *dodona* from Lima and Arequipa that are currently separated by approximately 600 km, without a record of species.

The evidence of connectivity between populations using different *Malesherbia* species, based on the individual admixture of alleles and shared haplotypes of *D*. (*A.*) *dodona*, allows us to infer a high dispersal capability in this species. Likely using a stepwise pattern, individuals keep an ongoing gene flow between populations along 1200 km.

### 4.3. Conservation of Suitable Areas

Desert areas separating the currently documented *D. (A.) dodona* populations are almost completely devoid of vegetation, representing potential barriers to the dispersal of flying insects, as recently reported for a leaf miner micromoth whose geographic range partially overlaps that of this butterfly [50]. High connectivity between populations despite potential barriers suggests that a given species could be less affected by anthropogenic modifications [51]. However, under the accelerated rate of habitat modification and destruction throughout the distribution range of *D. (A.) dodona*, its dispersal ability may not be enough to maintain population connectivity. Accordingly, habitats in which this butterfly and its host plants occur must be protected as a first step toward contributing to the conservation of the still insufficiently known biodiversity of the arid environments of the western slopes of the Andes.

## 5. Conclusions

*Dione* (*Agraulis*) *dodona* is an oligophagous species which uses at least seven different species of *Malesherbia* (Passifloraceae) as host plants within its distribution range on the western slopes of the Andes of Peru and Northern Chile. Such a distribution coincides with that of species located within the section *Malesherbia*, recorded as the only host plant group, suggesting that these are selected for oviposition, thus conditioning the geographic distribution of this butterfly. We did not find significant genetic differentiation between population samples from different host-plant species. Our results indicate high genetic exchange among populations between the different localities and host plants. It remains unknown how such a gene exchange is made among such geographically isolated populations located in the valleys of the Andes.

## Figures and Tables

**Table 1:** Results of the AMOVA testing for geographic structure in *Dione (Agraulis) dodona* populations. Localities were categorized into two groups: north (LIM + ARE + MOQ + TAC) vs. south (CH1 + CH2). For each level, the variance components (i.e., the variance explained by a given level) and the proportion of variance explained by the level in the global model are presented. Values in the table are averaged to two decimal places.

Source of Variation	Variance	Variation (%)	*F* Statistic	*p*
Among groups	0.18	13.5	0.13	0.1241
Among populations within groups	0.15	11.6	0.13	0.0000
Within populations	0.99	74.7	0.25	0.0000

## Data Availability

Data are contained within the article and supplementary material. Raw haplotype sequences are deposited in the BOLD Systems (http://www.boldsystems.org/, accessed on 18 July 2022) under the project BIGLE (005-22 to 009-22).

## Supplementary Materials

The following supporting information can be downloaded at https://www.mdpi.com/article/10.3390/insects13090819/s1. Table S1: Collection data for larvae of *D*. (*A*.) *dodona* analyzed in this study. Table S2: Summary of basic information of the eight amplified microsatellites, with repeat motifs; annealing temperatures (Ta); primer sequences; size range for PCR products; number of alleles per loci; and estimates for *F*_ST_, *F*_IS_, and expected (H_E_) and observed (H_O_) heterozygosity. Table S3: The *p*-values for the Hardy–Weinberg exact test, by population by loci. Table S4: Summary for genetic variability of populations of *Dione* (*Agraulis*) *dodona* based upon 6 microsatellite loci and 654 bp of the Cytochrome oxidase subunit I sequences. Populations: Lima (LIM), Arequipa (ARE), Moquegua (MOQ), Tacna (TAC), Chile 1 (CH1), and Chile 2 (CH2). Abbreviations: n, sample size; A, the average number of alleles per locus; %P, percentage of polymorphic loci; Ho, observed (average) heterozygosity; He, expected (average) heterozygosity; nh, number of haplotypes; Hd, haplotype diversity; p, nucleotide diversity. Asterisk indicates statistical significance (*p* < 0.05). Table S5: Parameters of genetic diversity for all *D. (A.) dodona* populations analyzed over the Western Andes slopes: total number of alleles (A), allelic richness (AR), expected heterozygosity (He), and observed heterozygosity (Ho). Only the four microsatellite loci without null allele signals were used for calculations. Table S6: Polymorphic sites (except ambiguous ones) at the COI gene for populations of *Dione* (*Agraulis*) *dodona* (*n* = 34). Nucleotides are numbered from 1 to 654. Each haplotype distribution is represented per locality and by the total number of individuals per haplotype. Abbreviations for populations are given in Supplementary Materials Table S2; periods represent an absence of the correspondent haplotype. Figure S1: Bayesian analysis of individuals of *Dione* (*Agraulis*) *dodona* based on microsatellite data. Likelihood values for each number of groups tested (i.e., the K value) with Structure 2.3. The empty circles represent the exact likelihood values for each run, with K ranging from 2 to 6. Filled circles and complete lines represent the absolute value of the approximate second derivative of the mean likelihood for each tested K value according to Evanno et al. [32]. Figure S2: Mismatch distribution analysis shows bimodal distribution (non-significant) in three populations of *Dione* (*Agraulis*) *dodona*: Lima (LIM), Arequipa (ARE), and Moquegua (MOQ).
