# Supplementary material for: Host Plant Variation and Lack of Genetic Differentiation in Populations of Dione (Agraulis) dodona Lamas & Farfán (Lepidoptera: Nymphalidae)
Source: Insects. 2022 Sep 8;13(9):819. doi: 10.3390/insects13090819 (PMC9506569; doi:10.3390/insects13090819)
Supplement: Supplementary file 1 [file insects-13-00819-s001.zip › insects-1847433-supplementary.pdf]

Supplementary material 1: Tables S1-S6

Table S1. Collection data for larvae of *Dione* (*Agraulis*) *dodona* analyzed in this study.

Country/Region/Locality	Altitute	Coordinates	Date DD/MM/YY	*Malesherbia* species
Peru / Lima / Canta	1720 m	11°33'6"S / 76°42'54"W	24/06/2021	*M. tubulosa*
Peru /Arequipa / Pacaychacra	1500 m	15º54'S / 72º33'W	24/07/2021	*M. tenuifolia*
Peru /Arequipa / Pacaychacra	1500 m	15°55'08.0"S / 72°32'59.8"W	24/07/2021	*M. fatimae*
Peru /Arequipa /Yura	2600 m	16°13'14" S / 71°42'14"W	21/04/2022	*M. angustisecta*
Peru / Moquegua /Torata	2090 m	17°06'30.2"S / 70°50'36.6"W	26/07/2021	*M. ardens*
Peru/ Tacna/ Chululuni	2080 m	17°20'44"S / 70°27'26.4"W	26/07/2021	*M. arequipensis*
Chile /Arica /Cuesta El Águila	1580 m	18° 29' 03" S / 69° 51' 52"W	10/12/2021	*M. auristipulata*
Chile/Arica/ Las Peñas	1580 m	18° 33' 08" S / 69° 46' 03" W	15/12/2021	*M. tenuifolia*

Table S2. Summary of basic information of the eight amplified microsatellites, with repeat motifs, annealing temperatures (Ta), primer sequences, the size range for PCR products, number of alleles per loci, and estimates for F_{ST}, F_{IS}, and expected (H_E) and observed (H_O) heterozygosity.

Locus	Repeat motif	Ta (°C)	Primer sequence 5'-3'	Size range	Alleles	F_{ST}	F_{IS}	H_E	H_O
Dmon1	(CA)15	52	F-CGAACCACAAGTTCCTGAGTT	295-387	4	0.230	0.010	0.514	0.509
			R-ACGCGTGGATGTAGACACAA						
Dmon2	(CA)9	52	F-TTCACATCAAGATCAATGACCAG	131	monomorphic	1.000	N/D	0.000	0.000
			R-CCGTATTGCACGATGTTCAC						
Dmon9	(GT)20	52	F-CAATTCACAATCTATTGTTTTTGTTTA	191-343	3	0.166	-0.361	0.196	0.267
			R-CAGTGAACGACGAGTGATCG						
Dmon10	(TG)12	52	F-CAGGCTACGCAAGGAGAATC	152-188	2	0.749	0.439	0.178	0.100
			R-CTAGCAGTGGCAACGATTGA						
Dmon13	(GT)15	55	F-ACAATAAAGGGCAGCACTGG	183	monomorphic	1.000	N/D	0.000	0.000
			R-GTGAAGGACGGGGTGAGTTA						
Dmon18	(CA)10	52	F-GGCTTCTTCAAGCTGACCAC	247-395	7	0.212	-0.072	0.482	0.516
			R-TGACAGTCAACATGTGTGTGC						
Dmon22	(ACAT)8… (ACAT)6	55	F-CACGCACCTTCTTTGTTTCA	177-211	2	0.135	0.488	0.369	0.189
			R-TCAATGACAGTGGACGAGGA						
Dmon27	(AC)14	52	F-GTGTATCCGTGTGCGCTCTA	276-296	3	0.250	0.481	0.404	0.210
			R-TCTTGCTTACGCGTGGACTA						

Table S3. P values for the Hardy–Weinberg exact text, by population by loci. * p<0.05

	LIM	ARE	MOQ	TAC	CH1	CH2
Dmon1	1.000	0.891	0.292	0.029	0.029	0.172
Dmon9	0.850	0.958	-	-	0.056*	0.576
Dmon10	-	0.236	0.055	0.213	-	-
Dmon18	0.557	0.560	0.159	0.577	0.284	0.655
Dmon22	0.187	0.725	0.020	0.134	0.072	0.351
Dmon27	0.045*	0.002	0.057	0.598	0.612	0.241

Table S4. Summary for genetic variability of populations of *Dione* (*Agraulis*) *dodona* based upon 6 microsatellite loci and 654 bp of the Cytochrome oxidase subunit I sequences. Populations: Lima (LIM), Arequipa (ARE), Moquegua (MOQ), Tacna (TAC), Chile1 (CH1) and Chile2 (CH2). n, sample size; A, the average number of alleles per locus; %P, percentage of polymorphic loci; Ho, observed (average) heterozygosity; He, expected (average) heterozygosity; nh, number of haplotypes; Hd, haplotype diversity; p, nucleotide diversity. Asterisk indicates statistical significance (P < 0.05).

Population	Microsatellites					COI						
										Tajima's	Fu and Li's	
	n	A	%P	Ho	He	n	nh	Hd	Pi	D	D	Fs
LIM	8	1.7	62	0.27	0.43	5	3	0.70	0.00245	-1.09380	-1.09380	-1,1133
ARE	10	2.2	75	0.33	0.34	5	2	0.40	0.00122	-0.97256	-0.97256	-0,95440
MOQ	10	1.8	62	0.32	0.43	9	2	0.22	0.00034	-1.08823	-1.18990	-1,28293
TAC	10	1.8	50	0.36	0.49	8	1	0.00	0.00000	-	-	-
CH1	9	1.6	62	0.40	0.44	6	1	0.00	0.00000	-	-	-
CH2	5	1.6	37	0.39	0.52	3	3	1.00	0.00512	-	-	-
Total	52	1.8	100	0.28	0.45	33	5	0.282	0.00097	-2.09252*	-2.94043*	-3,13553*

Table S5: Parameters of genetic diversity for all *Dione (Agraulis) dodona* populations analysed over the Western Andes slopes: total number of alleles (*A*), allelic richness (*AR*), expected heterozygosity (H_e) and observed heterozygosity (H_o). Only the four microsatellite loci without null allele signals were used for calculations.

**Locality**	**abbrev.**	***A***	***AR***	H_e	H_o
Lima	LIM	6	1.7	0.49	0.29
Arequipa	ARE	9	2.2	0.26	0.20
Moquegua	MOQ	10	2.5	0.54	0.38
Tacna	TAC	9	2.2	0.40	0.22
Chile1	CH1	**7**	1.7	0.44	0.33
Chile2	CH2	**7**	1.7	0.57	0.31
Mean (±sd)			2.0±0.8	0.39± 0.18	0.27± 0.16

Table S6. Polymorphic sites (except ambiguous ones) at the COI gene for populations of *Dione (Agraulis) dodona* (*n* = 34). Nucleotides are numbered from 1 to 654. Each haplotype distribution is represented per locality and by the total number of individuals per haplotype. Abbreviations for populations are given in Supplementary Materials Table S2; periods represent an absence of the correspondent haplotype; "x" represent presence of haplotype.

Haplotype Number	Freq.	Site no.									Haplotype per locality					
		12	17	47	80	161	185	284	413	605	LIM	ARE	MOQ	TAC	CH1	CH2
H1	1	G	A	G	T	A	A	T	A	G	-	-	-	-	-	x
H2	2	.	.	A	C	T	C	.	G	A	x	x	-	-	-	-
H3	29	.	.	A	.	T	C	.	A	A	x	x	x	x	x	-
H4	1	C	G	A	.	T	C	.	.	A	x	-	-	-	-	-
H5	1	.	.	A	.	T	C	A	.	A	-	-	-	-	-	x

Supplementary material 2: Figures S1-S2

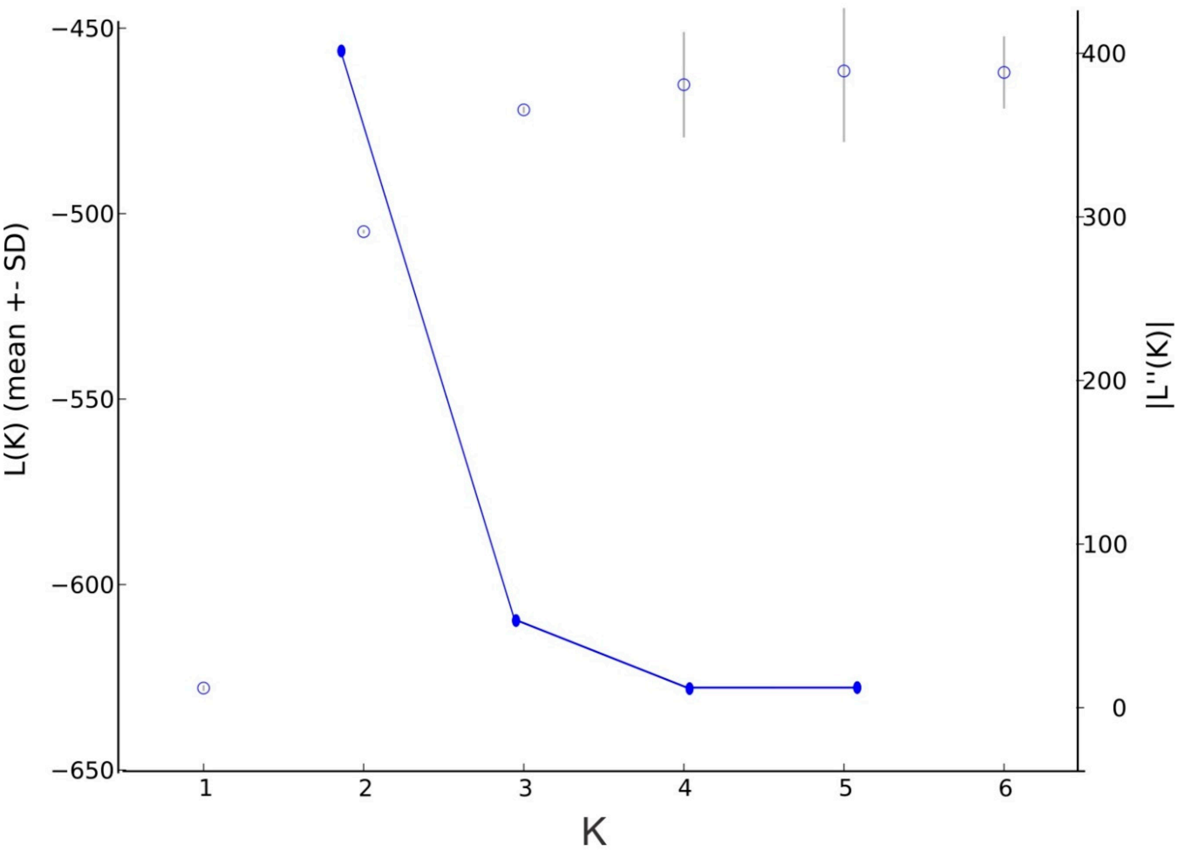

Figure S1. Bayesian analysis of individuals of *Dione* (*Agraulis*) *dodona* based on microsatellite data. Likelihood values for each number of groups tested (i.e. the *K* value) with Structure 2.3. The empty circles represent the exact likelihood values for each run with *K* ranging from 2 to 6. Filled circles and complete lines represent the absolute value of the approximate second deriva-tive of the mean likelihood for each tested *K* value according to Evanno et al. [32].

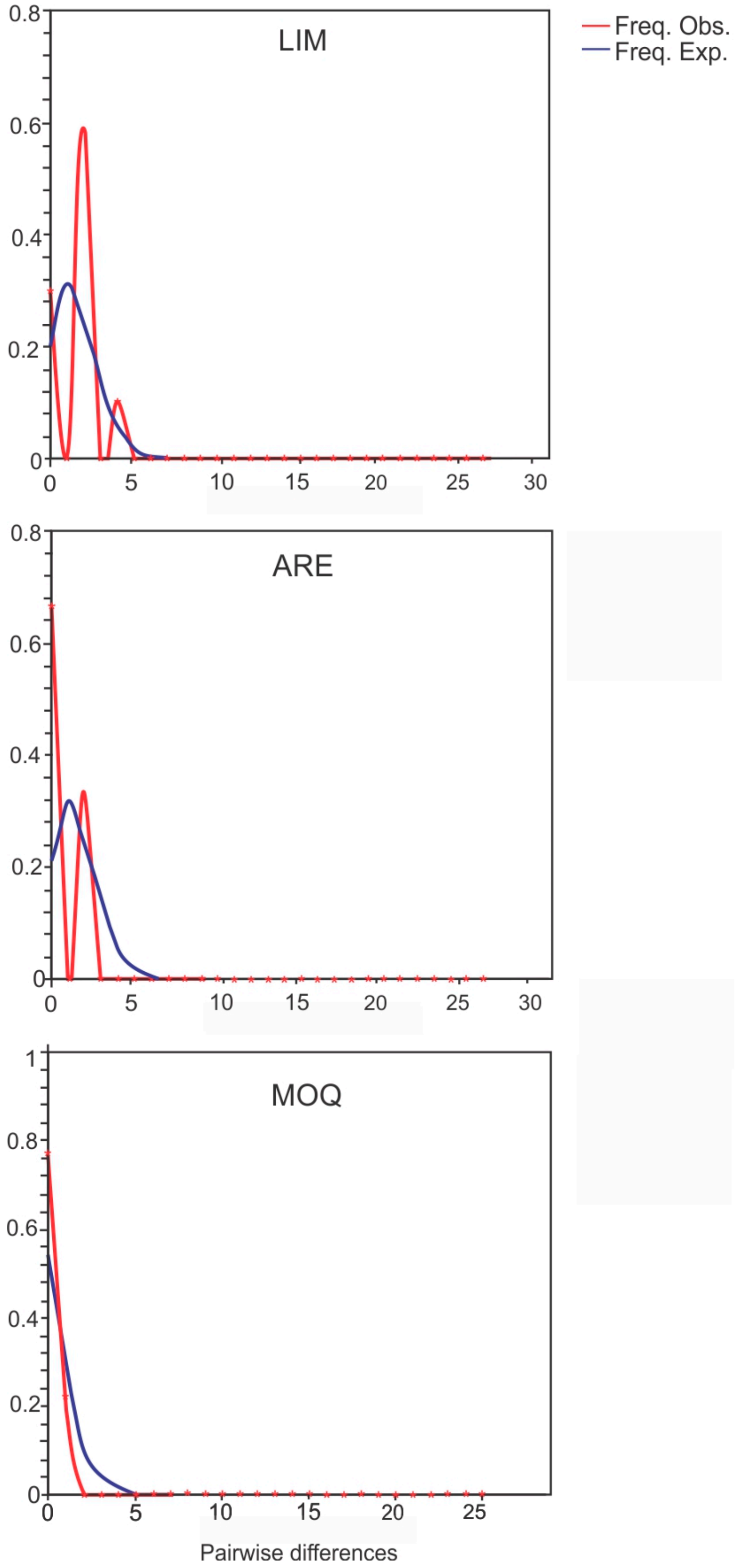

Figure S2. Mismatch distribution analysis shows bimodal distribution (non-significant) in three populations of *Dione* (*Agraulis*) *dodona*: Lima (LIM), Arequipa (ARE), and Moquegua (MOQ).
